# Short Historical Account of Dispensatories, or Pharmacopœiæ

**Published:** 1809-10

**Authors:** 


### Medical and Physical Journal.

VOL. XXII.] OCTOBER, 1809. [NO. 128.

*Printed for R. PHILLIPS, by W. Thorne, Red Lion Court, Fleet Street, London.*

### SHORT HISTORICAL ACCOUNT OF DISPENSATORIES, OR PHARMACOPŒIÆ.

IT is well known that the early physicians were the dispensers of their own medicines, that they were surgeons also, and prepared their own salves. Very soon, however, a part of this office devolved upon the nurses, or more immediate attendants on the sick, who, on that account, were called Θεραπευοι, or servants, from which we have the term *therapeutics*, for that branch of medicine which relates to remedies and the mode of exhibiting them.

We should remark on this occasion, that in proportion to the poverty and uncultivated state of a community, is the number of domestic servants in each family. Where manufactories are in their infancy, and the consumption of manufactured articles small, the sources of industry are proportionally few, and the support of a considerable part of the community is by the bounty or protection of the great. On this account, it will be less surprising, when we find a Roman citizen having in his family a Greek servant, who was expected to be sufficiently acquainted with the composition of well known remedies, to prepare, and even administer them to the household. If the case became urgent, the *medicus* was consulted ; and in more distinguished houses among the Romans, he was not uncommonly a domestic servant, probably either a Greek slave, or a freed man.

It will easily be seen, that in such a state of society, remedies would multiply almost in proportion as the practitioners were numerous. But this would be of little consequence, as long as the practice of each was confined to a single family. As, however, these warlike people became less disposed to spend their lives in camps, they became more sensible of the value of health, and even of life; and the celebrity of some individual physician would soon render his services more generally demanded.

Thus we find, at a very early period, that the gratitude of the great usually induced them to liberate their *medicus*; if he had been their slave, to admit him to their familiarities, and show every external mark of respect during life and after death. Such characters could not fail to attract the admiration of the public, and to induce such whose friends were in danger under disease, to apply for their assistance. Still, however, the different branches of medicine remained for a considerable time without any accurate distinction. The physician could easily avail himself of the remedies which the family dispenser or Θεραπευς, supplied, or direct others to be procured of which he might have a higher opinion.

From this we may easily trace the steps which rendered apothecaries and authorized dispensaries absolutely necessary, for the preservation of the public health. Every person who could boast possession of a receipt from a celebrated physician, might believe himself, or easily persuade the ignorant, that nothing more was necessary than to apply it when a similar disease occurred. We all know how difficult even the adept sometimes find it to distinguish diseases, and above all, how ready the ignorant are to confound symptoms with diseases, and to assimilate them all. Hence the possessor of a medicine for a disease will readily find applicants; and, as by the kindness of Providence, most diseases will ultimately, however slowly, yield to the powers of the constitution, the remedy would have the credit of the cure.

These possessors of secret medicines, whose whole object was to make the most of their traffic, appear to me to have availed themselves of the public credulity, in a manner which has escaped the notice of former writers, and tended to render pharmacy infinitely more expensive and complicated:—I should add, that though this is only a suggestion of my own, yet it is supported by so many and such conclusive evidences as appear almost a demonstration. When their remedies had proved successful to any public spirited and wealthy character, his wish to serve the interest of mankind would induce him to extend the benefits he had received as far as possible. This he conceived might easily be done by purchasing the receipt, and making it public. The medicaster, in his sale, would have two things in view; first, to preserve the dignity of his remedy, and next, if possible, to preserve his traffic after he had parted with his secret. To do this, before the invention of chemistry, two means would readily occur.

cur. The first would be to render his receipt so complicated, that all would admit the probability of virtues in some of the ingredients; or if not in some individually, at least, in the possible combination of some or of the whole. The next would be, to render it so expensive, as would justify the high value he had set upon them, and also to lead others to believe that it would be as cheap to continue to purchase as to make for themselves.

It was observed by a late ingenious physician, that the Theriaca and Mithridate had been changed at different times. The object of this was with some, if possible, to simplify so strange an assemblage; but with others, no doubt, to render it more complicated, and to boast the superiority of their own. In other compositions it is hardly possible to doubt, that the addition of precious stones and of the precious metals, could have no other object at first than knavery. The credulity of others was afterwards brought in aid; and in these, as on many other occasions, the best intentioned, laboured to support the dissolute, the profligate and designing. To such a cause as this we must impute the virtues at one time attributed to the *aurum potabile,* to the bezoar, to the sapphire, carbuncle, the hyacinth, pyropus, and amethyst, and even to the pearls that formed a part of the celebrated confectio cardiaca, which was so accurately prepared at Apothecaries' Hall, (and perhaps there only,) till the appearance of the Dispensatory in 1787.

I have dwelt so long on a subject apparently unimportant, to show the absolute necessity of some well authorized Pharmacopœia for the protection of the public, and to place honest men in a situation which may turn their integrity and industry to a proper account.

Let us now direct our attention to the progress of Dispensatories. The first of these, we may readily conceive, made a part of the cookery books, and were preserved among the stores of the housekeepers. In many of the modern languages, a *dispense* is the term used for the store-closet, and a *dispensaira* is the house-keeper. The analogy is evident enough, as the office was to regulate the *dispendium* of the various articles necessary in a family. Among these, broths, jellies, lozenges, candies and conserves, have always made part of the cookery book; and we shall find, in the first London Pharmacopœia, published by the College, a recipe for chicken broth, under the name of aquæ caponis. It is true, the air of mystery is preserved, for after the broth is boiled, it is to be ren-

 dered dered unpalatable by a variety of additions. Borage water, bugloss water, as well as their leaves, the leaves of roses and violets, and a small quantity of cinnamon; and to complete the absurdity, a portion of the crumb of bread; after which the whole is directed to be distilled in a glass retort.

In the same publication, the articles conserves and sugars conclude with a note, by which the reader is jocularly asked, why we should not leave the rest to confectioners, so called, and the ladies? *Confectionariis ut vocitant, & fœminis generosis quid non de cetero relinquamus?*

Among medical writers, the earliest physicians intermix their dispensatories with their other writings. The works attributed to Hippocrates, contain a great number of remedies; but we are by no means certain, which are to be considered as the production of the Father of Physic. Diascorides is a name familiar with every one; but we can hardly be said to have an intelligible Pharmacopœia of an earlier date than Celsus, and the greater part of his receipts are for external application.

A few centuries after, the Moors, being in possession of the most refined part of Europe, introduced what little they knew of chemistry into pharmacy, and from that time the system of mystery was improved to its highest. The technical language was unintelligible, and even the marks by which it was expressed were symbolical; of all this nothing now remains but the characters by which our weights and measures are designated; and as this is the only relic of barbarism with which medicine is chargeable, I could wish it were abolished with the rest.

From this time we can find nothing but a farrago of receipts collected from the Arabs or Moors, the ancient Greek writers, and some solitary formulæ, which by accident acquired celebrity at different times, and were aftewards recorded with the names of their inventors, or perhaps of the persons who were relieved by them. To these we shall presently refer, when we explore the sources of the first London Pharmacopœia; but it is impossible, on this occasion, to pass over a work published at Venice, in 1562, twenty years after a regular dispensatory had received a senatorial sanction at Nuremburgh. This work is entitled Mesve Greco. & Arab Medicorum quæ extant. It contains every thing written on disease and remedies, by the most celebrated Arabians, comprehending, besides Mesve, Avicenna, Albucasis, and Saladin; but the most curious curious thing that strikes the reader, is nearly an hundred closely printed pages, of canons for preparing the more simple remedies These are not indeed all imputable to Mesve, but to his various commentators, and particularly to Sylvius, and the publisher Andrew Marino.

By this, and many other instances, it will appear, that though the senate of Nuremburgh had adopted a dispensatory as early as 1542, yet the practice was by no means general. However, it is plain, from the preface prenxed to the first London Pharmacopœia, that the English College had been backward in so useful an undertaking. Perhaps it may not be so difficult to account for this backwardness. The College were probably more alive to the magnitude of the undertaking than other establishments, whose reputation was less considerable, and who consequently had less to apprehend from committing themselves too early. There is even reason to believe that their first publication was so much in request, and so long in agitation, as to encourage the boldness of some speculators, who taking advantage of the public eagerness for such a work, ventured to print it in a crude state. This, however unjustifiable, had the effect of forcing the College to execute their long meditated design, and exactly a century after they had received their charter from Henry, their first Pharmacopœia was published.

At this time it may appear strange, how the business of pharmacy could have been conducted without such an authorized work from the College. But the work itself, in some measure, explains that difficulty. In their preface they remark, that it may be thought unnecessary to add one to the number of Pharmacopœias, when there was scarcely a city, or university of any importance, that did not furnish one. Admitting, it is added, that many of these have satisfied themselves with what has been done by Mesve, Myrepsus, and Alexandrinus, still each city has chosen to have its own *Antidotarium.* The Romans, the Venetians, the Florentines, and they might have added among those included in the *quos non taceamus,* Bononians, Antwerpians, have all their Antidotaria; and why should not the Londoners possess theirs? For the want of this, they observe, that the apothecaries were often in doubts and uncertainties, whether they were to be directed by Cordus, Fernelius, or by what other authority. Hence we may suppose that each physician, when he wrote his prescription, was under the necessity of adding by what Dispensatory each composition was to be prepared; and

 this this might probably, in some instances, extend to even the MS. prescriptions of living authors.

In the first attempt at a London Pharmacopœia, we see many proofs of this. A great number of the compositions have the names of the inventors, or of the authors from whom they are immediately derived, attached to them. Mesve seems oftener referred to than any other. But Fernelius, Nicolas, Myrepsus, and Alexandrinus, have each of them their share. Even individual receipts are extracted from authors who have not written precisely on pharmacy, but on different branches of medicine. Galen's name stands at the head of several receipts: from Fallopius de Morbis Gallicis, we have an aqua aluminis magistralis, probably intended for gonorrhœa, or fluor albus in women. Fracastorius de Morbis contagiosis, furnishes a diascordium, and Langius an aqua bezoartica. There is also a philonium Persicum of Mesve, and philonium Romanum of Nicolas. Besides which, there are prescriptions which appear to have been productions of physicians, at that time members of their own body. In a copy of their first Pharmacopœia, preserved in the library at Bolt Court, are several heads of receipts marked in MS. as Dr. Mounteforde's and Dr. Mayerne's, the latter of whom was, at that time, first Physician to their Majesties.

Having given this general view of pharmacy, before the institution of Dispensatories, and their early infancy, I shall endeavour to show the manner in which they have gradually risen to greater perfection, and offer a hint or two towards the means of their further improvement.

I have never met with the original Dispensatory, as published at Nuremburgh; but the above library furnishes an edition, printed at Antwerp, in 1568, which is valuable, inasmuch as it affords us some account of the respectable author, to whom we are indebted for so valuable a performance, and also of the peculiar circumstances to which we owe the first authorized work of the kind.

Valerius Cordus, we are informed, was the son of a celebrated Physician. As in his epitaph he is called Simoisius Hessus, or the Hessian Archimedes, we may conclude he was a native of that part of Germany. Before he had finished his studies, and whilst engaged in a journey to Italy for that purpose, he made a transient visit at Nuremburgh. Here he was introduced to all the literary characters, and among the rest to the physicians. To the latter, during some some conversations on the subject of pharmacy, he produced a collection he had made of the best receipts he could procure from all the writers on medicine. It is very probable, that his father's experience had first suggested the idea, and instructed him in the selection. The physicians were so much struck with the sight, of a compact set of formulæ, that they requested him to give the apothecaries of Nuremburgh the benefit of his labours. Cordus, who probably had before conceived the advantage to be derived from an authorised Pharmacopœia, made many objections to offering a work so important to the health of the city, without the authority of the senate. The book was therefore presented to that body, who directed the college of physicians to peruse it in the presence of the author. When the college had signified their approbation, every apothecary was directed to prepare his medicines by the receipts it contained. And to this event we are indebted for the institution of public Pharmacopœias. We cannot help regretting, that the valuable youth, to whom we owe this first attempt, died soon after his arrival at Rome.

It would be extremely unjust to criticize the production of a mere student, brought into public light by the desire of the magistrates and faculty of a city of which he was a stranger. It may, however, be worth remarking, that though Cordus professes to give the compositions of the Arabians, who are known to have been the inventors of distillation, yet we have no mention of ardent spirits in any composition, excepting in a process for making an imperfect ether from spiritus vitrioli. In this place, the alcohol is called vinum ardens acerrimum ter sublimatum, by which I conceive must have been meant rectificatum, for the word distillation is only used when the process of procuring oil of vitriol, or essential oils, is described. Equal parts of this vinum ardens and oil of vitriol are directed to be put into a cucurbit, to the top of which an alembic is to be luted with much care. This was to be placed in a furnace, and half buried in cinders. The recipient was then to be luted, and the same quantity of ardent wine as was at first put in is to be drawn off. After this the remainder was to go through another distillation, and a separation of the oil from the water: the oil is directed to be preserved with great care, because you would get but a small quantity from a pound of the austere oil of vitriol, and that small quantity would soon evaporate. This oil was celebrated for dissolving tough humours of the liver and lungs, and also for preventing the formation of calculus in the kidneys and bladder, as well as for curing ulcers in the latter.

Besides this, we meet with directions concerning the mode of distilling essential oils and waters, but they do not properly make a part of the dispensatory.

About twenty years after this is dated the Cologne dispensatory, under the name of dipensarium usuale pro pharmacopœis inclytæ reipublicæ colonien ex commissione nobilium procerum, &c. Here we might have expected some improvement; and if we wanted only additions, we shall meet with enough. I know not whether the authors of this dispensary were the inventors of the aqua caponis; but in justice to the London College, whose Pharmacopœia appeared sixty years after, we remark, that their Aqua Caponis, if not equally efficacious, is a little more simple, and less cruel. The London College presume, that the animal will be killed and plucked before it is drawn, or *evisceratus*; but the Coloniens prescribe the manner of killing the animal, which is to be first teazed or hunted to death, *plurimum fatigatus*. The rest of the process is more elaborate, and the impediments somewhat more numerous. At the end, the author observes, that physicians might draw their swords to determine the virtues of capon water, with some other humorous remarks, which seem to refer to local circumstances and characters. On the whole, this work is destitute of order, and more complicated than the Nurembergh; it shows however a somewhat greater progress in chemistry, and the utensils as well as manipulations are more numerous, as well as more minutely described.

What the gradual progression of pharmacy was from this time until the appearance of the first London Pharmacopœia, I have neither had leisure nor industry to trace. But the form of this work is sufficient to show how extremely slow the progress of every art is, till it arrives at such a state as to catch the attention of the public, or to excite emulation among those who cultivate it. In Cordus's dispensatory I have remarked, that no notice is taken of simple distillation, but as an article of chemistry. In the London Pharmacopœia, the number of distilled waters amounts to 170, among which are the aq. Bursæ pastoris—Brassicæ—Scabiosæ et Spermatis ranarum; substances, none of which can be expected to yield any thing to the still. It is but justice to remark, however, that in this list, we meet with one very powerful and almost unmanageable remedy in the common mode of using it by infusion, this is the tobacco plant, which cannot fail I should should conceive to yield an essential oil, that might be turned to some account.

All the spirituous waters are also compound, that is, distilled from more than one ingredient besides the spirit. Though ardent spirits were not unknown, yet many of these waters, particularly the aq. hysterica, afterwards brioniæ compositus, are directed to be distilled from wine. There is a prescription for an aqua vitæ, ad usum pauperum; this is distilled from equal parts of sweet wort and the dregs of wine, with anniseed, in an alembic and refrigeratory. Among the compound waters is a receipt for usquebach, which is properly speaking a tincture, and the only one I have met with in the whole Pharmacopœia.

Medicated wines and vinegars follow; but in this, as in Cordus's dispensary, the most bulky part of the work is made up of syrups, confections, under the name of electaries and lohocks; pills, and unguents.

How much soever a late ingenious and much lamented physician may have been disposed to ridicule the theriaca and confect. damocratis, these were simple and unexpensive preparations compared with some others in this first Pharmacopœia. The antidotus magna Mathioli adversus venena et pestem, has a list of 126 articles, among which are several compounds, as the species cordialium temperatarum. Spec. diamargareton, and the aq. bezoartica, comprehending each a great number, and every expensive article; as castor, mosch, ambergris, all the precious stones, together with the two precious metals.

It would be very unfair in these days to examine critically the various preparations in a pharmaceutic work nearly 200 years old; but it is impossible not to mark the haste with which the whole is got up. Besides numberless other inaccuracies in the aqua bezoartica brevis Langii, we have in one part Cinnam. dr. ijß. Carioph dr. ij. And in another part of the same prescription, Cinnam. dr. iij. Carioph dr. ijß. This can no otherwise be accounted for than by the great hurry in which the work was produced, in order, as soon as possible, to supersede the spurious production.

The date of the next London Pharmacopœia which I have been able to collect, is 1651. This in many respects comes nearer to the present form. It contains a chapter of tinctures. They are few in number, and excepting the Tinct. Castorei, of little efficacy. There is a Tinct. Fragorum, a Tinct. Scordii, and a Tinct. Theriacæ, made of spirits distilled from Cretan or Canary wine, vinegar, the two long long compositions of mithridate and theriaca, digested in a bath heat. If Mathiolus's antidote is omitted, the number of heavy preparations is in many respects increased; and the aqua caponis still holds its place.

Dr. Powell has favoured us with a catalogue of the different Pharmacopœiæ published by the College, according to their dates. From such diligence, and such means of information, we cannot doubt the accuracy of the result. But the prefaces to those of 1650 and 1677, somewhat obscure the subject to those who have not the opportunity of seeing the rest.

The duodecimo edition of 1651 begins with remarking, "Ante duos, supra sex, lustra, annos visum est collegio nostro edere suam pharmacopœiam." If we take the lustres at five years, this will make a period of thirty-two years, which deducted from fifty makes eighteen, the date of the first Pharmacopœia published by the College.

The edition of 1677 begins, "Ante tres supra decem lustra visum est, &c." This, according to the former computation, would reduce the period to which the writers allude, to 1632, which agrees with one of Dr. Powell's dates. Both these have a dedication in the same words to King James. This dedication is copied from the first Pharmacopœia of 1618, at which times James the 1st. reigned.

The editions of 1627, 1632, and 1639, must have been published during the reign of Charles the 1st. That of 1650 during the inter-regnum and before the protectorate; and that of 1677 during the reign of Charles II.

The last has indeed an inscription between the title and the old dedication, in which the College place their Pharmacopœia under the protection of the reigning monarch, but still the dedication to James is retained. There is an edition of 1681 in the library at Bolt Court; but this was evidently only a republication, without any alteration, and probably without the notice of the College, as not only the preface and dedication are copied from 1677, but also the College list.

The alterations in all these are so gradual and so little worth notice, that it is probable no attention was thought necessary after the first edition, to require the royal mandate for their reception among the apothecaries. In the materia medica, indeed, the progress towards simplicity seems to have been retrograde; for besides retaining all the disgusting component parts of several animals, and their excrements, the edition of 1677 has an addition of *secundina*: of what animal no mention is made. In all these the preface varies but little, and no copy that I have seen after the first contains the Royal proclamation.

From this to the appearance of a new Pharmacopœia, a longer period elapsed than between any of the former. This is less to be wondered at, when we consider that the progress of improvement in natural knowledge had now become such as very much to increase the difficulty of compiling a work which had received no considerable improvement for nearly a century. But if the undertaking was arduous, fortunately, there were not wanting men the best adapted for it. This edition has the Royal proclamation; it is inscribed (without a dedication) to George the 1st. and the preface, which we shall presently notice, was new.

This Pharmacopœia (of 1720) was published under auspices the most favourable. The venerable name of Sir Hans Sloane appears as president, nor was he at that time in his wane. Only the year before he had been chosen to that honourable office, and he could boast as his coadjutors the elegant and accomplished Mead, the industrious and candid Frend, and Jurin, who laboured so hard to reconcile the world to the reception of small-pox inoculation. Among the licentiates indeed the name of Sydenham appears, but not the great man to whom the world owes so much: nor indeed a single name that, as far as we can judge, could assist the College in their labours, or even adorn their list.

But no assistance was wanted! We may venture to assert, that so complete a work of any kind was not produced so early in the 18th century. The preface, with only a single exception, and in that I am incompetent to judge, appears a perfect specimen of latinity, modesty, and genuine urbanity. The arrangement is as complete as in the knowledge of those days could have been expected. The reference to their predecessors, to the state of the shops under the long neglect of amending the Pharmacopœias, delicate; and most of all, the manner in which the surgeons of that date are introduced in a style highly complimentary and worthy of the professors of each branch.

Twenty-five years afterwards appeared another Pharmacopœia. The list still retains most of the same names, but alas, many of them were mere names. Sloane had then reached his 80th year, Mead had exceeded the usual term of mortality, Frend was no more, and Jurin's attention had been distracted by controversy. The stately Hulse, the profligate Barrowby, the pedantic and noisy Brown had succeeded. What could the modest Heberden do, at that time the last of the list, and probably but just torn torn from his alma mater, the scene of calm repose or of learned industry? It is impossible not to be struck with the different language in which two works, proceeding from the same sources, are ushered into the world. "Our ancestors, says the venerable Sloane and his coadjutors, seem most happily to have consulted their own dignity and the public service, when they took upon themselves the care of publishing a Pharmacopœia, an undertaking not less laborious than necessary; and since, by the favour of the sovereign, they were appointed the inspectors of medicine, not only in this city but throughout England, they would have been wanting in their public duty, had they omitted such an office. This office, undertaken with zeal, they accomplished with industry, &c."

Let us hear the language of their successors. "It pleased the President and the College to subject the London Pharmacopœia to a new inquisition, which indeed the safer and more compact mode of prescribing among the more celebrated physicians seemed to require." Then follows a mode of reminding the reader of their privileges, without any expression of gratitude to the sovereign, as on the former occasion. After which, without any acknowledgement of the labours of their predecessors, all the follies of antiquity are brought to open day-light, and we are almost taught to believe, that the framers of the former Pharmacopœia, some of whom were then living, had been the inventors of antidotes against poisons, of which they were constantly afraid; or that, like old women, they consulted oracles, dreams, and astrologers, superstitiose et aniliter, petierunt ex oraculis insomniis & commentis astrologis."

It must be admitted, however, that the work was materially improved; and if the larger compositions are retained, an apology is made for yielding, in some instances, to customs which they recommend to posterity to lay aside.

The next revision of the Pharmacopœia was in 1787. To notice the preface of this edition would be superfluous; though long, it cannot be called tedious; though every where classical, it is no where pedantic or obscure; and whilst the errors of more remote antiquity (prisci) are slightly hinted at, a just praise is given to those more immediate predecessors, (antecessores) who near half a century before, "summa diligentiæ et judicii laude id expleverunt munus quo nunc fungimur. By this time, as the writers remark, chemistry had begun, with the discovery of a new science, to invent new names. It has been thought thought by some that the College were premature in adopting them; and I well recollect, that my friend Austin, who was at that time a chemical lecturer, inclined to that opinion. It would ill become one, whom chemistry has for a long time left at an immeasurable distance, to offer an opinion on this subject. Thus much may, however, be remarked, that the same objections to altering names might be extended to the terms of Majestery, Regulus, Panacea, and Catholocon. Let us take only a single example: Hiera picra, the very sound of which appears now ridiculous, is from a language admitted to be the most musical of any in the world. It was however thought right to change it for Latin, and tinct. sacra usurped its place; this is now tinct. aloes composita. These alterations, necessary as they may seem, are trifling, compared with others still more important. In short, to retain the obsolete names, and such they would be to the rest of the world, would be to assume an air of mystery unbecoming a liberal art.

If it is urged that the learning of new names adds to the difficulties of acquiring a science already too much for the life of man, it is easily answered, that to the rising generation they are not more new than the older, and their elders will rarely have occasion to accuse others of want of candour, if they do not immediately fall into the new language.

This new language is introduced by an art which was formerly more immediately connected with medicine, but which is now branched into so many forms, that the principles only can be expected to be retained by physicians, or by apothecaries practising in the present state of the profession. It is said that mistakes have occurred from the change. This is highly probable; but if the late proposal of writing always at full length were adopted, this difficulty could scarcely occur.

Some have objected to the language in which Pharmacopœias and extemporaneous prescriptions are written. In Portugal, and I believe in Spain, it is always in the language of the country. However, a general language is by many thought more desirable, as intelligible in every part of the world. The London College has done wisely in authorizing a translator, which obviates every difficulty of that kind in their Pharmacopœia. Still there is one objection to the use of Latin, which may not immediately occur to every reader, and that is, the natural wish of every writer, in that language, to render his sentences as purely classical as possible. This will sometimes produce an an unnecessary obscurity, principally by transpositions, which should be as much as possible avoided. In the preface it may be admissible to indulge the effusions of a good taste, and a happy recollection of classical phrases: but I am mistaken if even this wish has not formerly betrayed the writers into a figurative mode of expression, hardly justifiable. It is worth remarking, that the preface to all the Pharmacopœias, excepting those of 1745 and 1787, contain an allusion to the same figure from the *ars poetica*. The first, in its *epilogus*; the others, in their preface. The *epilogus* is so full of figures, that the reader may, at first, be at a loss to know which of them refers to *ad incudem denuo revocavit*: besides, that revocare *ad incudem*, is a phrase scarcely admissible. The preface to the edition of 1720, though perfectly chaste in all other respects, seems chargeable with the same want of propriety in the use of this term. The recent publication preserves the original phrase, *incudi reddere*.

Nothing can be more pleasing than the recurrence of certain sounds from the favourite work of a favourite author; and, on a disputed passage, I shall not be so hardy as to offer an opinion,* especially as the propriety of every other part of the preface is a fair presumption, that the authors have judged correctly in this.

It may be a little flattering to an English reader, that in all the Pharmacopœias I have had leisure to examine, and as far as my knowledge enabled me to judge, those of London are the most correct in their latinity. I shall only instance one of the irregular words, as these are the most certain means of detecting the scholarship of the writers. In the Paris Pharmacopœia, published in 1758, we have the word *ostreum*, evidently arising from the recollection of the neuter plural *ostrea ostreorum*. In the Swedish Pharmacopœia of 1787, we have *ostrearum* testæ, from the recollection of the singular fæminine *ostrea*. The English Pharmacopœia of 1746 and of 1787, have, as might be expected, avoided both these errors.

---

* If I recollect rightly, Rollin, in his introduction to the *Belles Lettres*, objects to the passage, because a lathe has no connection with a mallet or forge. *Et male tornatos incudi reddere versus.* Bentley, I believe, has *ter natos*, and some other Critic, *formatos*.

Those who have seen a gentleman artisan turn a piece of metal, may easily comprehend the force of the expression, for if once the cutting instrument is directed wrong, every attempt at recovering the error is fruitless, and the only remedy is, to commit the metal to be hammered over again, and to begin the work afresh. The allusion seems to follow, that instead of patching a lame verse, it is better to make a new one. I before remarked, this was not the case with any Pharmacopœia during the 17th century. *Ad incudem revocare*, appears also an improper association of words.

In the first London Pharmacopœia, the arrangement of weights is from the grain to the pound. This was continued in every subsequent edition till the publication of 1745. In this, the arrangement begins with the pound, which rendered necessary the detail of the different weights in use in the kingdom. Perhaps the original plan might have some advantage. It begins by fixing the grain according to the standard of weighing money, which is I believe the same throughout every part of the world. From this, without noticing any other kind of weights, it was easy for the College to ascertain their own. In doing this, however, they proceed to the scruple, the drachm, and pound, and give the symbols of each. Here is a manifest impropriety in intermixing such characters with Roman weights. The notice of these characters was first omitted in the Pharmacopœia of 1745. I cannot help wishing that they were omitted in all extemporaneous forms. There is at least a great impropriety in using such characters to distinguish scruples, drachms, and ounces, whilst we assume a classical affectation in marking the numbers of each by the old Roman characters. These last are not only much more inconvenient than the Arabic, but more liable to be mistaken if only written in the common running hand. In the first Pharmacopœia also, there was some attempt at introducing the Roman measures; but they accord very ill with those in modern use. Thus we find the cochleare, cyathus, hemina or cotyla, the libra, the sextarius, and congius. These refer to the uncia and drachma, without any mode of fixing a standard for any. The scale is rendered still more obscure by the sextarius being rated according to the Roman measures, at a pound and half, and the congius at six sextarii, or nine pounds and half. In the editions of 1650 and 1677, the hemina and cotulus are omitted, but no standard is proposed till that of 1720, in which the wine pint is specified as the pound measure. The Pharmacopœia of 1745, omits also the cochlea, and adds the P or M, to distinguish weight from measure. The same plan is pursued in the edition of 1787. In this, for the first time, attention is paid to the thermometrical heat and specific gravity. The recent publication has the advantage in all these respects; the introduction of the word octarius cannot be objected to as a substitute for the libra, and the addition of the minimum is highly important. As, however, the symbols are no where used in the work, I could almost wish they had been omitted. But this rather belongs to my last proposal, of offering a few hints whenever another impression shall be again thought necessary.

The new work appears to possess much merit, and to be liable to as few objections as can be expected in such an undertaking. Respecting weights, I can see no reason why we should not confine ourselves, like most experimental philosophers, to grains altogether, or at least omit all the intermediate divisions from them to ounces or even pounds. By these means we might avoid all the Roman names, as well as technical characters. In the measures the word congius is objectionable, as marking a Roman measure different from our gallon. I can see no objection to gallo or gallonium, as the Pharmacopœa of 1745 had pinta for our pint.

The great difficulty in the construction of a Pharmacopœia is the chemical arrangement, and the accurate and ready recollection of the various affinities. Chemistry is a science much too extensive to be accurately followed by any other professional man. In one respect too, it places the most experienced practitioner in the most unfavourable view, for whilst he is improving a practical art, which can only be imperfectly learned by the closest attention during a long life, he must be inattentive to those discoveries which are daily arising in a science depending so much on experiment. Hence the student of to day, in chemistry, will be better informed than the veteran who could instruct him in the more important branches of his profession. Add to this, we are all too apt to value ourselves on new acquisitions, and if we feel our inferiority in some respects, to retort the same on others. Hence the office of the College, in forming a new Pharmacopœia, must be full of difficulties, some of which it will not be in their power to meet, but by the assistance of others. On these accounts, I cannot help doubting, whether it might not, for the future, be better to leave this task to others, and to offer only their approbation to such as they have reason to believe is the most unobjectionable. It has been said, that both Edinburgh and Dublin exceed the London College in the facility with which they complete their Pharmacopœias. Nothing is more probable. They are both the seats of Universities, where encouragement is given to professors, and where to teach, which is the best means of learning, is the highest honour to which some of their members aspire. Besides this, they have more leisure, because the line of professional engagements is more confined.—These are only a few of the difficulties which attend such an undertaking. Let us then reflect how easy it is to find fault, how dignified to overlook errors if they exist, and how small must be the merit of bringing before the public view such as the authors themselves are ready to admit.

*Observations*

